# Supplementary material for: Humoral and Cellular Response after mRNA Vaccination in Nursing Homes: Influence of Age and of History of COVID-19
Source: Vaccines (Basel). 2022 Mar 2;10(3):383. doi: 10.3390/vaccines10030383 (PMC8948814; doi:10.3390/vaccines10030383)
Supplement: Supplementary file 1 [file vaccines-10-00383-s001.zip › vaccines-1573259-supplementary.pdf]

Membership of the investigators of the SeroVAC study

File S1: Gema Fernández-Álvarez, Diana Sánchez-Migallón, Beatriz Benavente, David Pozuelo, Julia Salmerón, Daniel Fernández, Alfredo Carrillo, Pedro Medina, Rosa Arnal from Nursing Homes and Long-Term Care Facilities Support Unit, Dirección Asistencial Norte, Gerencia Asistencial Atención Primaria Madrid; Juan Ignacio González-Montalvo, Francesca Argentina, Ana Merello de Miguel, Montserrat Barcons Marqués, Blanca Chaparro Jiménez, Irene Moreno, Cristina Resino from Service of Geriatrics, Hospital General Universitario La Paz; Mª Pilar Bernáldez, Ángel González, Lorena Vicente, Noemi Anguita, Carmen Sáez , Rafael Bielza from Service of Geriatrics, Hospital Universitario Infanta Sofía; Javier Gómez-Pavón, Margarita González, Irene Díaz, Pablo Victoria, Esperanza Fernández from Service of Geriatrics, Hospital Universitario Cruz Roja
